# VirE2: A Unique ssDNA-Compacting Molecular Machine 

**DOI:** 10.1371/journal.pbio.0060044

**Published:** 2008-02-26

**Authors:** Wilfried Grange, Myriam Duckely, Sudhir Husale, Susan Jacob, Andreas Engel, Martin Hegner

**Affiliations:** 1 Centre for Research on Adaptive Nanostructures and Nanodevices, Trinity College Dublin, Dublin, Ireland; 2 National Centre of Competence in Research Nanoscale Science, Institute of Physics, Basel, Switzerland; 3 M. E. Müller Institute for Structural Biology, Basel, Switzerland; 4 Novartis AG, Basel, Switzerland; 5 Rowland Institute at Harvard, Harvard University, Cambridge, Massachusetts, United States of America; 6 Friedrich Miescher Institute for Biomedical Research, Basel, Switzerland; University of British Columbia, Canada

## Abstract

The translocation of single-stranded DNA (ssDNA) across membranes of two cells is a fundamental biological process occurring in both bacterial conjugation and *Agrobacterium* pathogenesis. Whereas bacterial conjugation spreads antibiotic resistance, *Agrobacterium* facilitates efficient interkingdom transfer of ssDNA from its cytoplasm to the host plant cell nucleus. These processes rely on the Type IV secretion system (T4SS), an active multiprotein channel spanning the bacterial inner and outer membranes. T4SSs export specific proteins, among them relaxases, which covalently bind to the 5' end of the translocated ssDNA and mediate ssDNA export. In Agrobacterium tumefaciens, another exported protein—VirE2—enhances ssDNA transfer efficiency 2000-fold. VirE2 binds cooperatively to the transferred ssDNA (T-DNA) and forms a compact helical structure, mediating T-DNA import into the host cell nucleus. We demonstrated—using single-molecule techniques—that by cooperatively binding to ssDNA, VirE2 proteins act as a powerful molecular machine. VirE2 actively pulls ssDNA and is capable of working against 50-pN loads without the need for external energy sources. Combining biochemical and cell biology data, we suggest that, in vivo, VirE2 binding to ssDNA allows an efficient import and pulling of ssDNA into the host. These findings provide a new insight into the ssDNA translocation mechanism from the recipient cell perspective. Efficient translocation only relies on the presence of ssDNA binding proteins in the recipient cell that compacts ssDNA upon binding. This facilitated transfer could hence be a more general ssDNA import mechanism also occurring in bacterial conjugation and DNA uptake processes.

## Author Summary

The importation of genetic material into cells is a common and fundamental mechanism occurring in bacterial conjugation, DNA uptake, and *Agrobacterium* plant infection and is, for instance, responsible for antibiotic resistance spread. Previous studies suggested that this process relied only on the activity of complex molecular machines pumping the single-stranded DNA (ssDNA) into the recipient cell. Here, we show that proteins provided by the pathogenic organism and translocated prior to the arrival of ssDNA into the recipient cell also play a fundamental role. These proteins not only bind to ssDNA to protect it but also rearrange ssDNA into a compact helix, thus generating a contractile force that pulls the DNA into the host. Interestingly, the production of mechanical energy occurs solely through the free-energy gain during the binding of VirE2 to ssDNA without the need for an external source of energy, such as nucleotide hydrolysis.

## Abstract 3

Combining single-molecule techniques and biological assays, the authors show that VirE2 proteins could mediate the active translocation of*Agrobacterium*'s genetic material into plant cells.

## Introduction

Agrobacterium tumefaciens is a Gram-negative pathogenic bacterium able to transfer and integrate up to 150,000-bases-long single-stranded DNA (ssDNA) into the infected cell nuclear genome [1]. In *Agrobacterium* pathogenesis, the sequence of ssDNA to be transferred (T-DNA) and the genes encoding the virulence (Vir) proteins required for transfer of T-DNA into the host are localized on a large plasmid called the tumor-inducing plasmid [2]. Some virulence proteins have a function in the bacterium, namely the 11 VirB proteins and VirD4, which compose the Type IV secretion system (T4SS) machinery. T4SS exports T-DNA and effector proteins out of the bacterium [3–5]. The effectors are proteins, which are synthesized in the bacterium but exert their function in the recipient cell. The export signal of the effector proteins is localized at their C terminus and is recognized by VirD4 [6]. Among the effectors, the relaxase VirD2 binds covalently to the 5′ end of the ssDNA. The combined action of the three NTP-binding/hydrolysing proteins VirB4, VirB11, and VirD4 has been proposed to energize the transfer of the proteins and VirD2-T-DNA through the T4SS [7]. How the T-DNA then crosses the plasma membrane of the host remains unknown, but the effector protein VirE2 might be involved. In vitro, VirE2 was shown to form channels, which transport ssDNA, and VirE2 was hence proposed to mediate transfer of T-DNA through the eukaryotic plasma membrane [8–10]. VirE2 is a necessary, multifunctional protein [11] and another important function of VirE2 is to bind cooperatively T-DNA in the host cytosol. The interaction of VirE2 with T-DNA mediates its import into the nucleus. As evidenced by scanning transmission electron microscopy (STEM), the VirE2–ssDNA complex consists of a helical structure in which 19 nucleotides are bound per VirE2 monomer [12]. This structure prevents exonuclease degradation in vitro [13]. Moreover, recent in vitro experiments demonstrate the microtubule-guided transport of such DNA–VirE2 complexes [14].

Using single-molecule technology, we measured the binding properties of VirE2 to ssDNA, and we suggest here that VirE2 binds to ssDNA nucleotides in a zipper-like mode. This property was confirmed biochemically with the ability of the VirE2 protein to bind to a shorter oligonucleotide than its footprint of 19 nucleotides. We also show that cooperative VirE2 binding compacts the ssDNA against high loads (50 pN), which could, in vivo, help to actively pull the T-DNA into the recipient cell. Using cell biology detection techniques, VirE2 was localized at the plant cell periphery, an ideal localization for VirE2-mediated pulling of the incoming T-DNA. Altogether, a combination of very different techniques allowed the emergence of a completely new view on T-DNA transfer energetics upon translocation into the host plant cell.

## Results

### Force-Dependent Polymerization Kinetics on ssDNA

**Figure 1:**
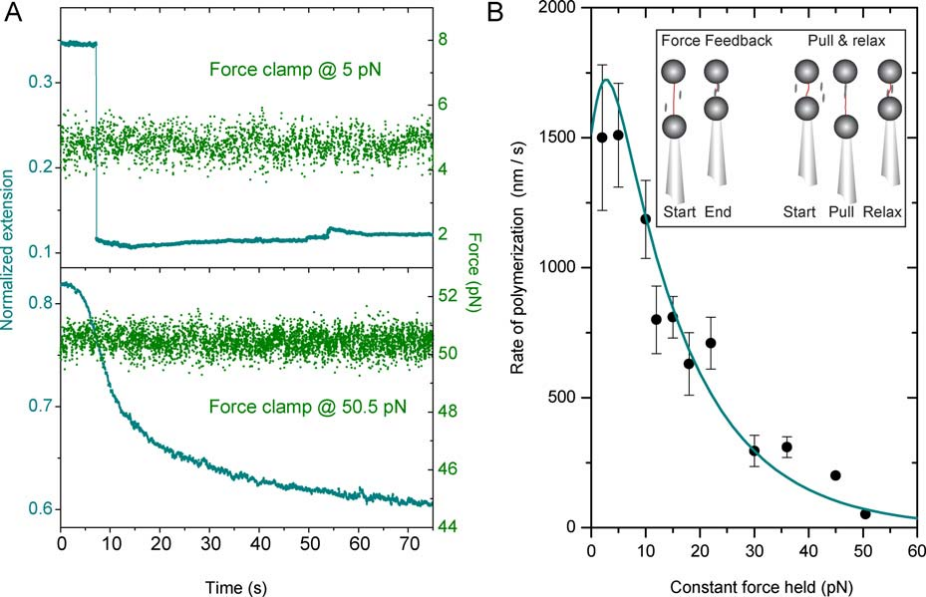
Force-Feedback Experiments on ssDNA in the Presence of VirE2 Proteins (A) Force-feedback time traces of a single ssDNA molecule compacted after VirE2 injection. The blue solid line is the extension (normalized to the contour length of ssDNA), the green line is the constant force signal. Upper panel: force-feedback at 5 pN. Lower panel: force-feedback at 50.5 pN. (B) Polymerization rate *k*(*f*) of VirE2 proteins as a function of the applied tension on the ssDNA template. At each force, *k*(*f*) is expressed in nm/s (A). An analysis presented in the Text S1 [section: Rate of polymerization (experimental determination)] allows the rate originating from a single polymerization front to be determined. The observed force-dependence of *k*(*f*) is overlayed by a theoretical model using known structural parameters of both ssDNA and VirE2-ssDNA complexes (solid line; see main text section: Local binding mode, and Text S1 section: Rate of polymerization (theory)]. The inset shows optical tweezers modes used. Force feedback: the pipette displacement is controlled by the servo-system to maintain a constant tension on the ssDNA during protein injection. Pull and relax: the force is recorded upon movement of the pipette (extending and relaxing the filament in the presence of proteins) without feedback.

Binding of VirE2 to ssDNA was studied using different optical tweezers modes (Figure 1B, inset). First, the VirE2 binding rate was determined at a pre-set force that was kept constant by a feedback system (force-feedback experiment, [Figure 1B, inset]; VirE2 concentration of 20 μg/ml, 330 nM). Polymerization of VirE2 dramatically affected the length of the tethered ssDNA (Figure 1A). From a detailed analysis of the time traces [Figure S1, showing a plot where the transition takes place; Text S1, section: Rate of polymerization (experimental determination)], we found a value of (1510 ± 200) nm/s (*n* = 5) for the polymerization rate originating from a single nucleation site (5 pN). For the 4,502-bases-long DNA and taking 19 as the number of nucleotides bound per VirE2 monomers (as determined by scanning transmission electron microscopy) [12], this yields a binding rate of ∼10 VirE2/ms at a VirE2 concentration of 20 μg/ml (5 pN). These force-feedback experiments were performed at different forces. For forces ≤22 pN, the normalized extension at full polymerization was found to be about 0.11 ± 0.02 (*n* = 21). Compaction also occurred even when the ssDNA was forced to remain in an extended form (50.5 pN; Figure 1A). At this force, the polymerization rate was found to be considerably slower (∼ 50 nm/s, Figure S1). Force-feedback experiments performed at high forces (>22 pN) yield a normalized extension at full VirE2 coverage of 0.66 ± 0.05 (*n* = 11), much longer than the one observed at low force (about 0.1) (Figure 1A). This 6-fold difference in normalized extension (observed at full coverage) indicates that VirE2 filaments adopt a different global structural arrangement depending on the preset force. This point will be discussed in details below (section: Global Structural Arrangement of ssDNA upon VirE2 Binding).

### Local Binding Mode

**Figure 2:**
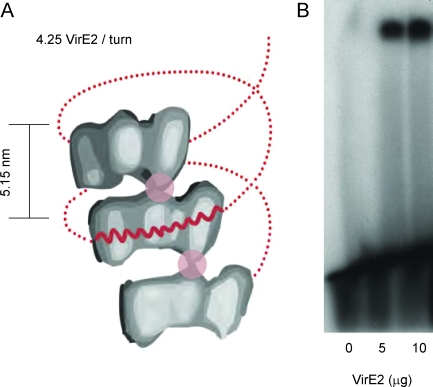
Local Binding Mode of VirE2 on ssDNA and Helical Structure of VirE2-ssDNA Filament (A) Schematic representation of the VirE2-ssDNA helical structure (adapted from [18]). Only three individual VirE2 monomers are shown. The approximate location of the ssDNA helix is shown in red (dots). The overall VirE2-ssDNA structure is stiffened up by strong axial interactions between the VirE2 proteins (pink circles). The binding of VirE2 monomers to ssDNA is such that the distance between adjacent ssDNA-phosphates of the backbone is 0.41 nm projected along the long axis of the protein (thick red line). (B) The formation of nucleoprotein complex between VirE2 and 5 pmoles of a 12-mer oligonucleotide was analyzed by radioactive gel shift assay. Lane 1: 12-mer without VirE2. Lanes 2 and 3: 12-mer with, respectively, 5 μg and 10 μg of VirE2. Upon interaction of VirE2 with the 12-mer, a higher-mass complex can be detected in the upper part of the gel (lanes 2 and 3). This indicates the binding of VirE2 to the 12-mer. The addition of double the amount of VirE2 leads to the formation of double the amount of complex (lane 3), showing that the formation of the higher mass complex is VirE2 dependent.

Force-feedback measurements at different forces allow the force dependence of the polymerization rate *k*(*f*) (originating from a single polymerization front, see Figure S1) to be determined (Figure 1B)*.* Using the Arrhenius law, *k*(*f*) is described by *k*(*f*) *= k*_0_ exp(*-<w*(*f*)*>/k*_B_ *T*), where *<w*(*f*)*>* is the work produced by VirE2 per locally bound single nucleotide [15], *k*_0_ isthe rate at zero force, *k*_B_ is Boltzmann's constant, and *T* is temperature. In a local model, *<w*(*f*)*>* is approximated to *f* (*L_SS_<*cos*θ> – L_V_*), (Text S1, section: Rate of polymerization (theory), and Figures S2 and S3, showing a detailed analysis of the model), where *L_V_* (*L_SS_*) is the DNA base-to base backbone distance in the presence (absence) of VirE2. From structural data, a value of 0.7 nm is found for *L_SS_* [16]. In a freely jointed chain (FJC) model, *<*cos*θ>* follows the Langevin formula [17], yielding an analytical expression for *k*(*f*). The local model gives a good description of the experimental data when the base-to-base distance of the VirE2-bound ssDNA *L_V_* equals 0.41 nm (Figure 1B and Figure S3). This value (0.41 nm) is estimated from electron microscopy (EM) studies (assuming the ssDNA to lie concentrically within the protein helix [18], Figure 2A) and is in good agreement with a statistical analysis of the compaction steps (Figure S4, probability density function (PDF) analysis of the time trace at 50.5 pN). The good description of the experimental data by the local model suggests that VirE2 monomers bind one nucleotide at a time in a zipper-like motion and that the probability of binding a nucleotide is site-independent, a prerequisite for the local model. Such a model predicts that VirE2 could bind stably to less than 19 nucleotides. Experimental proof was provided by a gel-shift assay (Figure 2B), which demonstrated that VirE2 can bind a 12-bases-long oligonucleotide. Finally, the good description of the force-feedback measurements (Figure 1B) by the local model suggests that the base-to-base distance of ssDNA bound to VirE2 is force independent**.**

### Coverage of ssDNA in the Presence of VirE2

**Figure 3:**
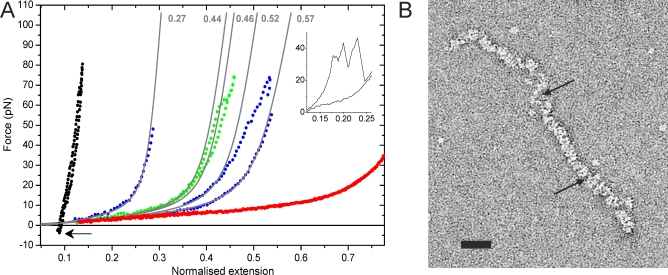
Fully Covered ssDNA-VirE2 Filaments Consist of Stiff Rods (A) Force versus extension curves of fully coated (black), partially coated (blue, green) ssDNA-VirE2 filaments, and uncoated ssDNA (red) in assembly buffer at low protein concentration. Extension curves are normalized to the contour length of ssDNA measured prior to exposure to VirE2. Three typical ssDNA-VirE2 curves were recorded within ∼120 s. Gray lines are extensible FJC fits; the numbers (top) indicate the fraction of uncoated ssDNA. Abrupt jumps in the force-extension curve indicate cooperative release of VirE2 domains (blue curve and sawtooth pattern in inset). Multiple pull-and-release cycles produce a single rigid helical domain (black curve) that resists compression forces of ∼3.5 pN (obtained by reversing the direction of pulling [buckling force, arrow]). (B) ssDNA–VirE2 complexes observed by negative-stain EM showing multiple nucleation sites leading to different VirE2-ssDNA helical domains, which are not in register (arrows indicate the separation between helical domains). Scale bar: 30 nm.

Standard force-versus-extension curves (pulling and relaxing the tethered DNA molecule without any feedback; “pull and relax”) (Figure 1B, inset) at lower protein concentration (6 μg/ml, 100 nM) were recorded (Figure 3A). These curves show the progressive compaction of bare ssDNA (red) as coverage with VirE2 proteins occurs, up to a state where the filament adopts a stable conformation (black). This final conformation (for which subsequent pulls did not noticeably change the shape of the force-versus-extension curves) yields an average compaction factor of 9.7 ± 2.0 (*n* = 15). Previous EM studies have reported a compaction factor of 11.9 for a perfect VirE2-ssDNA helical structure (Text S1, section: Length reduction upon protein binding). This suggests that the final state we observe (also confirmed by distance-clamp experiments, Figure S6) corresponds to a conformation where the VirE2 proteins rearrange into a helix (Figure 2A). As seen in Figure 3A, the final state (black curve) is extremely stiff (as compared to ssDNA). Curves recorded at intermediate stages of polymerization (green, blue) can be fitted with a FJC model considering the ssDNA compaction factor upon VirE2 binding of 11.9, the persistence length of bare ssDNA and a normalized contour length *l* = λ*l_ssDNA_ +* (*1 -* λ)/11.9 (0 < λ < 1), where *l_ssDNA_* is the normalized contour length of ssDNA (Figure 3A, gray lines). Therefore, partially coated ssDNA-VirE2 filaments (blue and green curves) exhibit two domains. First, the flexible, uncoated ssDNA of length λ*l_ssDNA_*, and second the almost nondeformable, fully VirE2-coated domain of length (*1 -* λ)/11.9.

Through the sequential binding and subsequent release of VirE2 proteins from bare ssDNA molecule (red curve), the final helical conformation is obtained (black curve). In Figure 3A, the intermediate state of polymerization (blue curve, normalized extension of about 0.2) shows the detachment of a large amount of VirE2 proteins at ∼50 pN (yielding a decrease in the VirE2 coverage, i.e., an increase in the fraction of bare ssDNA in the filament from λ = 0.27 to λ = 0.46). When the force applied to ssDNA was relaxed, VirE2 molecules bound to ssDNA again, achieving a more stable coverage, since almost no VirE2 was driven off upon restretching of the DNA–protein complex up to 70 pN (green curve). These findings correlate nicely with the binding mode of VirE2. VirE2 is a non–sequence specific ssDNA binding protein, and the interaction of VirE2 with ssDNA (at a low protein concentration of 6 μg/ml) leads to multiple nucleation sites. This yields a number of different VirE2-ssDNA helical domains, which might not be in register (i.e., yielding a nonperfect helical structure over the whole length of the ssDNA, Figure 3B). When the VirE2-ssDNA filament is pulled, short VirE2 domains seem to progressively detach from the ssDNA molecule. When the tension is relaxed, VirE2 proteins bind again. Subsequent pulls yield an increase in the average length of VirE2 helical domains, which then resist higher forces (green curve). The final state therefore corresponds to an extremely stable conformation in which no VirE2 release from the filament is observed when proteins were removed from the fluid chamber.

### Mechanical Properties of VirE2 Filaments (Full Polymerization)

The fully polymerized nucleoprotein complex was unusually stiff (Figure 3A, black curve). From the critical force for buckling |*F*_B_| ∼ 3.5 pN (obtained while compressing the filament; Figure 3A, arrow), we estimate a persistence length (|*F*_B_|*l* ^2^/4π^2^ *k*_B_ *T*) [19] of ∼14 μm, about 4 orders of magnitude larger than that of bare ssDNA [20]. Because binding of ssDNA to VirE2 in a zipper-like way requires some initial protein flexibility, the high stiffness measured in the final (fully covered) VirE2-ssDNA filaments suggests that VirE2, the DNA, or both are stiffened by their interaction. A similar increase in stiffness upon DNA binding has been observed for other ssDNA binding proteins such as RecA [21]. As mentioned in the Text S1 (section: Mechanical properties of ssDNA-VirE2 filaments: Helix model), a mechanical model consisting of a pure helical structure gives a value of 110–2,200 nm for the persistence length, much lower than reported here. This difference could be attributed to the presence of axial interactions along the protein helix (reported by EM studies [18]) that could considerably stiffen the structure (Figure 2A, circles).

### Global Structural Arrangement of ssDNA upon VirE2 Binding

For force-clamp experiments performed at low forces (≤22 pN, Figure 1), the value for the normalized extension at full coverage was estimated to be at 0.11 ± 0.02 (value obtained from a total of 21 experiments performed between 2 and 22 pN). This value for the normalized extension is in good agreement with that of EM studies for a perfect helical arrangement (0.084 or 1/11.9 [18]), suggesting that the helical VirE2-ssDNA structure can even form against loads up to ∼20 pN.

This helical conformation was not achieved when performing force-feedback experiments at >22 pN. For these forces and at full coverage, the normalized extension was found to be 0.66 ± 0.05 (estimated from a total of eight experiments performed at 30, 36, 45, and 50.5 pN). This normalized extension corresponds to an average base-to-base distance of ssDNA (projected along the direction of the applied force) of 0.46 ± 0.04 nm (Figure S5, showing typical force versus extension curves of both ss- and double-stranded (ds) DNA), in close agreement with that found from EM studies (0.41 nm, Figure 2A) [18]. From this result, we deduce that the rearrangement of the VirE2-ssDNA filament into a helix (Figure 2A) cannot proceed against large forces and that the normalized extension reduction observed at forces >22 pN corresponds to the sole binding of VirE2 on ssDNA.

The small discrepancy between the expected value and the experimental observation, although significant, can be attributed to the large footprint of VirE2 (19 nucleotides) as well as the possible loss of cooperativity at high forces.

The local model (Figure 1B and Figure S3) was shown to give a good description of the force dependence of the rate of polymerization. However, this model only considers the zipper binding mode of VirE2 to ssDNA and does not take into account the rearrangement into a helical structure. This suggests that the helical rearrangement is much faster than the local binding of VirE2 to ssDNA. Hence, the binding of VirE2 to ssDNA is the rate-limiting step of the overall polymerization process and dominates the kinetics.

Note finally that we did not observe any compaction of the ssDNA molecule for force-clamp experiments performed at low protein concentrations (<1 μg/ml). This correlates with gel-shift retardation experiments (Figure S7), which demonstrate that binding of VirE2 to 170-bases-long ssDNA occurs over a small range of protein concentration without intermediate bands.

### Localization of VirE2 in the Plant Cell

In vivo, the VirE2 protein exerts its role in the plant. It is sufficient to express the VirE2 protein in the plant to restore full virulence: transgenic plants expressing VirE2 allow efficient T-DNA transfection by nearly avirulent *virE2*-null-*Agrobacterium* [22]. If the VirE2 proteins accumulate at the periphery of the plant, then the interaction of VirE2 and ssDNA would not only protect the T-DNA from exonuclease degradation but also greatly facilitate the import of the T-DNA thanks to the capability of VirE2 to work against large forces when binding to ssDNA (see above).

**Figure 4:**
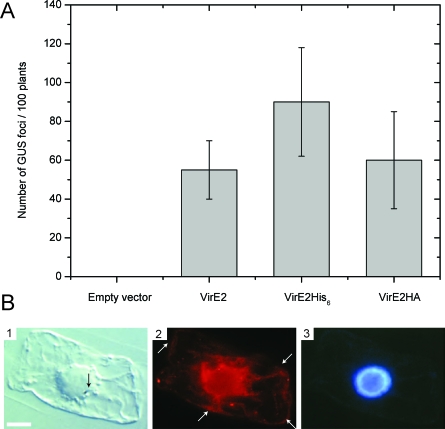
Localization of Active VirE2 Protein Transiently Expressed in Tobacco BY-2 cells (A) Tobacco plants were transformed with empty CAMBIA vector or CAMBIA vector allowing expression of VirE2, VirE2HA, or VirE2 His_6_. These transgenic plants were then used for transient T-DNA transfer assay. In this assay, the *Agrobacterium* strain lacking the *virE2* gene was used to infect the plants and the VirE2 protein in the plant, if active complements the missing VirE2 activity of bacterium-origin. The T-DNA is also carrying a reporter gene which transfer can be detected by the appearance of blue spots (GUS) on plants. One can observe that all the VirE2 variants used in this study are active as they allow complementation of the *Agrobacterium* strain lacking the VirE2 gene. (B) (1) Tobacco BY-2 cell transformed by gold particles bombardment observed in Nomarski. The gold particle (located in the nucleus) is indicated by an arrow. The scale bar represents 10 μm. (2) Localization of VirE2. VirE2 is present around the nucleus, in cytoplasmic strands, representing a soluble, cytoplasmic pool of protein also visible at the cell periphery (white arrows) and in a few cytoplasmic spots. (3) DAPI staining of the nucleus of the transformed cell.

Because localization of VirE2 protein originating from the bacterium has proven to be extremely challenging and has so far not yielded a usable result, we chose to use the fact that, when VirE2 is expressed in the plant, it is active. Hence, we transiently expressed VirE2HA in tobacco BY-2 cells. VirE2HA is a biologically active fusion (Figure 4A) and was used to perform immunofluorescence experiments. Figure 4B demonstrates the localization of VirE2 around the nucleus, at the cell periphery, in cytosolic strands, and in a few cytoplasmic spots. This non-nuclear cytoplasmic localization is supported by VirE2-GFP localization (also an active fusion protein when expressed in plant cells**,** S. Gelvin, personal communication). On the contrary, β-glucuronidase (GUS)-VirE2 fusion protein was reported to localize in the nucleus [22]. This controversial results might be explained by the fact that the GUS-VirE2 fusion protein mimics the conformation of VirE2 when bound to ssDNA and hence get imported into the nucleus (V. Citovsky, personal communication). Also, it is widely accepted by the community that the VirE2-ssDNA complex already forms in the host cytoplasm, allowing subsequent nuclear import of the nucleo-protein complex into the nucleus [2]. Hence, there must be some free VirE2 proteins in the host cytosol, which is consistent with our localization data (Figure 4B).

## Discussion

**Figure 5:**
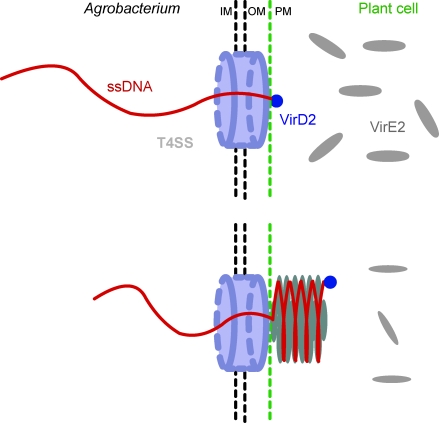
Model of T-DNA Translocation into the Plant Host Cell Transferred ssDNA-VirD2 is exported out of the bacterium through a Type IV secretion system (T4SS, light blue) and reaches the host cell cytoplasm. There, the cooperative arrangement of VirE2 into a helical structure actively pulls the incoming T-DNA into the plant cell cytosol. Additionally, the large speed of polymerization (∼1 μm/s) mediates a fast protection of the T-DNA at the very moment it enters the host cytosol. IM: inner membrane, OM: outer membrane, PM: plasma membrane.

The *Agrobacterium* pathogenesis mechanism allows for the efficient transfer of long ssDNA molecules into eukaryotic cells [2]. The VirE2 protein is involved in this process by protecting the ssDNA from nuclease degradation and by mediating nuclear import [2]. Here, based on new experimental findings, we propose that VirE2 is an effector that is transported into the host cytoplasm at an early stage to actively pull the T-DNA into the host and protect it from nuclease degradation from the very first moment it enters the cell. In a first step, a single VirE2 protein binds to T-DNA as it enters the plant cell. This binding, occurring in a zipper-like motion, is mainly limited by thermal fluctuations of T-DNA. In a second step, the fast cooperative binding of VirE2 facilitates the formation of a helical structure and actively pulls T-DNA into the plant cytosol (Figure 5). This model has indirect assumptions. First, VirE2 and T-DNA should not interact in the bacterium, even though they are both synthetized there. Indeed, in *Agrobacterium*'s cytoplasm, VirD2-T-DNA and VirE2 do not interact, and VirE2 only binds to the T-DNA once it is in the plant cytosol [23]. Second, the VirE2 protein should be present at the site of entry of the T-DNA, namely at the periphery of plant cells. This was evidenced by immunofluorescence experiments (Figure 4B), suggesting that VirE2 is properly localized to assist T-DNA pulling as it enters the plant cytosol. Finally, the interaction between VirE2-bound ssDNA and the rigid microtubule network could provide an anchor point that would facilitate the VirE2 mediated-force transduction at an early stage of the translocation process [14].

According to our model, which identifies VirE2 as an essential factor that pulls T-DNA into the plant cytoplasm, the free energy released upon the formation of the nucleoprotein complex allows VireE2 proteins to work against large forces, which might be required to translocate T-DNA into the host (see below). The production of mechanical energy occurs solely through the free energy gain during the binding of VirE2 to ssDNA without the need for an external source of energy, e.g., nucleotide hydrolysis. To our knowledge, this is the first time that a glimpse at forces involved in ssDNA translocation into the recipient cell is obtained. Their magnitude compares to forces produced by dsDNA translocating molecular motors (see, e.g., [24]). Other competing mechanisms might tend to pull the DNA back out of the host cytosol. For instance, during conjugation, pili can retract after binding to the host cell [25]. Moreover, during DNA transfer into the host, the Type IV pilus of Neisseria gonorrhoeae can undergo a series of extension and retraction cycles, generating retraction forces up to a few tens of pN [25]. Thus, binding of a protein to the transferred ssDNA to form a complex that prevents recoiling of the ssDNA in the T4SS by such forces would be a great advantage.

Tato et al. have proposed that the coupling protein TrwB of the Escherichia coli R388 conjugative system acts as an ATP-driven ssDNA transporting molecular motor [26]. This analogue to VirD4 is located at the bacterial inner membrane and is thought to pump ssDNA through the Type IV secretion channel. Considering the short persistence length of ssDNA (∼0.7 nm) and the large distance between the coupling protein and the host membrane (at least 30 nm [27]), just pushing the flexible ssDNA through the T4SS would be inefficient. Transfer would be facilitated if it were also actively pulled through by VirE2 present in the host.

Single-molecule experiments have shown that the “final” VirE2-ssDNA helical filament obtained is a very stable and stiff structure. Washing the complex with buffer without VirE2 protein does not destabilize the complex. But in vivo, the uncoating of the filament is necessary for the integration of the T-DNA into the nuclear genome of the recipient cell. Hence, the question is how the rigid VirE2-ssDNA complex is freed from VirE2. Indeed the very tight interaction between VirE2 and the ssDNA and between VirE2 molecules seem to need a specific mechanism of degradation to remove the VirE2 protein. It was shown recently that VirE2 is specifically targeted for degradation by the VirF-containing Skp1-Cdc53-cullin-F-box complex for proteolysis [28]. The critical role of proteasomal degradation in *Agrobacterium*-mediated genetic transformation was also evident from the inhibition of T-DNA expression by a proteasomal inhibitor. In summary, our findings and these data correlate nicely and explain why such a specific degradation mechanism would be needed.

The unique mechanism that *Agrobacterium* exploits to translocate any ssDNA molecule has paved the way for genetic engineering of plants and fungi but also offers novel possibilities for gene transfer into mammalian cells [2]. However, the *Agrobacterium* pulling mechanism proposed here might be more general. It does not rely on VirE2 but needs the following: (i) an ssDNA binding protein compacting ssDNA upon interaction and (ii) occurrence of this single-strand binding (SSB) activity only in one compartment. In bacterial conjugation and DNA-uptake processes, SSB proteins are also present and might have an important funtion. For instance, the SBB homologs (YwpH) accumulate preferentially at the cell poles of B. subtilis [29]. Hence these proteins could be, as is VirE2, capable of generating a force without external source of energy and pull the ssDNA into the recipient compartment.

## Materials and Methods

### Purification of VirE2-His6 proteins.

VirE2-His_6_ proteins were expressed in E. coli and purified as described in [30], with the addition of glycerol (final concentration 20% w/v) to the sample buffer (50 mM NaH_2_PO_4_, pH 8, 300 mM NaCl) before storage of the protein at −80 °C.

### DNA handles.

Two types of DNA handles were prepared and used either for force-feedback (type A) or pull and relax (type B) experiments. Type A: DNA molecules were prepared by PCR amplification (Taq DNA Polymerase, Roche, http://www.roche.com) of the pTYB1 plasmid (7,477 bp) [New England Biolabs (NEB), http://www.neb.com] using 5′-Thiol- TGG TTT GTT TGC CGG ATC AAG AGC −3′ and 5′- TCC TAA GCC AAC AAT AGC GTC CCA-3′ as forward and reverse primers, respectively. The 4,927-bp PCR fragment was digested with *Hind*III (NEB). Finally, the main fragment was end-filled with Klenow Exo- (NEB) with one dATP and one biotin-14-dGTP (Invitrogen, http://www.invitrogen.com), yielding a 4,502-bp-long dsDNA. Type B: DNA molecules were prepared by PCR amplification (Expand Long Template PCR System, Roche) of the pPIA plasmid (15,071 bp) using 5′-thiol-TAT CGT CGC CGC ACT TAT GAC TGT-3′ and 5′-TAT GTC GAT GTA CAC AAC CGC CGA-3′ as forward and reverse primers, respectively. The resulting 14,107-bp PCR fragment was digested with *Eag*I (NEB). After digestion, the longest fragment (13,883 bp) was end-filled with Klenow Exo- (NEB) with two dGTPs and two biotin-14-dCTPs (Invitrogen).

DNA molecules were covalently coupled to 2.17-μm amino-modified beads (Spherotech, http://www.spherotech.com) using sulfo-SMCC (Sigma) as a cross-linker [21].

### Optical tweezers.

The experimental apparatus for optical tweezers experiments has been described [31]. DNA beads were trapped by the laser and the free biotinylated DNA end was attached to a 2.20-μm streptavidin bead (Spherotech), which was held by suction on a micropipette. The bead-to-bead distance was determined from both the movement of the micropipette (controlled with a closed-loop piezoelectric element) and the deflection of the laser producing the optical trap (monitored by a two-dimensional, position-sensitive detector). The pipette bead was moved away from the trapped bead at a constant velocity of 0.8 nm/ms. At this rate, complete force-extension curves were recorded within a few seconds. Forces were obtained from the direct measurement of the change in light momentum flux [31]. All signals (distance, force) were low-pass filtered at 159 Hz. Force curves were measured in assembly buffer (50 mM NaH_2_PO_4_, pH 8.0, 150 mM NaCl, and 5% w/v glycerol). To obtain ssDNA molecules, dsDNA was exposed to 150 mM NaOH. Subsequently, the chamber was rinsed with assembly buffer and VirE2 proteins were injected. Prior to injection, proteins were centrifuged at 14,000*g* for 20 min. The supernatant was kept at 4 °C and injected at a protein concentration ranging from 6 to 20 μg/ml in assembly buffer. Forces were monitored in a constant VirE2 flow. Experiments were performed at room temperature.

### Force-clamp operation mode.

The force-clamp mode uses a digital “P”(proportional gain)-like feedback that runs at 150 Hz (taking into account the time for the acquisition, some CPU time for the calculations, and communication with the different instruments). In details, the feedback works as follows: if the change in force |Δ*f*| is smaller than 0.7 pN, we do not feedback at all; for larger changes in force, the pipette if moved by ±5 nm (|Δ*f*|≤ 2 pN) or Δ*f* × 7 nm (|Δ*f*| > 2 pN). During a force-clamp operation, the data are only recorded and plotted when |Δ*f*|≤ 0.7 pN. In that case, an additional ∼6 ms is required to process the different routines of the software.

### Gel shift of 12-mer oligonucleotide.

The oligonucleotide 5′-ACA TTG ACC CCT-3′ was radioactively labeled at the 5′ terminus by incubating 100 pmoles of oligonucleotide with 20 units of polynucleotide kinase (Roche) and 30 mCi of ^32^P γ-ATP (Pharmacia) for 30 min at 37 °C. The amount of incorporated radioactivity was measured using a TRI-CARD 2100 TR Liquid Scintillation Analyzer. Five pmoles (5,000 cpm) of the 12-nucleotides-long ^32^P 5′-labeled oligonucleotide were added to the VirE2 protein in 50mM NaH_2_PO_4_, pH 8, 300 mM NaCl, and the reaction was incubated on ice for 1 h. The mixture was then loaded on a native, 10% acrylamide gel and run in 0.25× TBE at 100 mV for 2 h at 4 °C. The gel was dried and exposed on a Kodak x-ray film. See Figure 2B.

### Gel shift assay of 170-bases-long ssDNA.

See Figure S7 and [30] for details.

### Generation of the constructs pCAMBIA-VirE2His_6_ and VirE2HA.

To clone VirE2H_6_ into pCAMBIAmod [32], the entire open reading frame (ORF) of pET-VirE2H_6_ [8] was amplified by PCR at 43 °C. A *Bam*HI site was added at the 5′ terminus using the primer 5′- CGC GGA TCC TTT AAC TTT AAG AAG GAG ATA TAC-3′ and a *Pst*I site was added to the 3′ terminus using the primer 5′-AAG ACG TCC TCA GTG ATG GTG ATG GTG ATG AAA GC-3′. The PCR product was cloned into pGEMT (Promega), cut with *Bam*HI and *Pst*I, and cloned into pCAMBIAmod that had been digested with the same enzymes, resulting in pCAMBIA-VirE2H_6_. pCAMBIA-VirE2HA was generated by digesting pCAMBIAmod with *Bam*HI and *Xba*I and inserting the VirE2HA gene extracted from pcDNA3.1-VirE2HA (see below) with the same enzymes.

Cloning of pcDNA3.1-VirE2HA was performed using the primers 5′-TCA TGG ATC CAC CAC CAT GGA TCT TTC TGG CAA TGA GAA A-3′ (adding a *Bam*HI site and the Kozak sequence on the 5′ of *Vir*E2) and 5′-ACT CTC TAG ATC AAG CGT AAT CTG GAA CAT CGT ATG GGT AAA AGC TGT TGC TTT GGC T-3′ (adding an hemaglutinin (HA) tag to the 3′ terminus of *Vir*E2 as well as an *Xba*I site) were used to generate VirE2HA by PCR amplification of the *Vir*E2 gene using pET- VirE2H_6_ as a template [8]. The PCR product was cut with *Bam*HI/*Xba*I and ligated into pcDNA 3.1 (Invitrogen) cut with the same enzymes. The resulting construct was named pcDNA3.1-VirE2HA.

### Generation of the constructs pCAMBIA-VirE2His_6_-int and VirE2HA-int.

For production of transgenic tobacco plants expressing VirE2 or mutants and to prevent expression of VirE2H_6_ and VirE2HA in *Agrobacterium*, an intron of potato *ST-LSI* [33] was inserted into pCAMBIAmod VirE2H_6_ and VirE2HA as a *Bam*HI/*Bgl*II fragment. The resultant plasmids were named pCAMBIAmod VirE2H_6_-int and VirE2HA-int. The plasmids were subsequently electroporated into electrocompetent *Agrobacterium* strain GV1301 (pPM6000) cells using a GenePulser (Biorad) at 2.5 kV, 200 Ω, 25 μFd.

### Generation of transgenic tobacco plants.

Transgenic plants expressing VirE2H_6_, VirE2HA were obtained by transforming tobacco (SR1) leaf discs with *Agrobacterium* GV1301 (pPM6000, pCAMBIAmodVirE2H_6_-int/ VirE2HA-int). Control plants were generated by transformation with the empty vector pCAMBIAmod. The selection was performed on Murashig and Skoog (MS) medium supplemented with BAP (4 μM), naphthalene acetic acid (NAA) (0.5 μM), cefotaxime (500 mg/l), timentin (150 mg/l), and hygromycin (20 mg/l). Individual plants were regenerated, and five plants from each category were transferred to soil for seed production. WT-VirE2 expressing plants were obtained from the laboratory of Andrew Binns [34].

### Assays for determining the efficiency of transient transfection of VirE2 minus *Agrobacterium.*

Seeds from transgenic plants (VirE2HA, VirE2H_6_) were sterilized and allowed to germinate on MS medium supplemented with hygromycin (50 mg/l). Fourteen-day-old seedlings were infected with diluted *Agrobacterium* GV1301 (pPM6000E, pCAMBIA 2201; *Agrobacterium* strain where the *virE2* gene has been deleted), cocultivated for 48 h to an optical density of 1, followed by extensive washing with MS medium [35]. For the last wash the medium was supplemented with timentin (150 mg/l). The histochemical GUS staining was performed as described [35]. Virulence was quantified as GUS positive spots per 100 seedlings.

### EM.

ssDNA fragments (M13) were incubated with VirE2 as described in [30] (Figure 4B).

### Particle bombardment of tobacco BY-2 cells.

Tobacco BY-2 cells were plasmolyzed on MS-agar plates with 0.25 M mannitol/sorbitol (Merck) for 3 h. DNA of pCAMBIA-VirE2HA and pCAMBIA-GFP was precipitated on 1-μm-diameter gold particles (Biorad). The particles coated with DNA were bombarded on the plasmolyzed BY-2 cells with a PDS-1000/He Biolistic Particle Delivery System (Biorad).

### Immunofluorescence localization of VirE2HA in BY-2 cells.

VirE2HA protein was transiently coexpressed with green fluorescent protein (GFP) after particle bombardment of tobacco BY-2 cells with plasmids pCAMBIA-VirE2HA and pCAMBIA-GFP. GFP was used as a positive marker for transformation. After 16 h recovery, the cells were fixed for 1 h in 3.7% paraformaldehyde (Sigma) in MSB/Gly buffer (50 mM Pipes, pH 6.9, 5 mM EGTA, 1 mM MgCl_2_, 2% glycerol). The cells were then washed three times with MSB/Gly buffer and deposited on polylysine-coated slides (polylysine L, Sigma). The cell wall was digested for 5 min with the following mix of enzymes from Yakult Honsha (Pectolyase 0.02%, Macerozyme 0.1% and Caylase 0.3%) diluted-10 fold in digestion buffer (25 mM MES, pH 5.5, 8 mM CaCl_2_, and 600 mM Mannitol). The cells were permeabilized with 0.1% Triton (Merck) in PBS (phosphate-buffered saline) for 5 min. Unspecific binding of antibody was prevented by incubation of the cells with 5% normal goat serum (Calbiochem). The rat monoclonal anti-HA antibody (Boehringer) was diluted 1:100 and the reaction carried out overnight at 4 °C. After washing the cells in PBS, the secondary antibody (goat anti-rabbit TRITC, Jackson Immuno Research Laboratories), was added at 1:30 dilution for 1 h at room temperature. DAPI (4′, 6-diamidino-2-phenylindole, Calbiochem), a nucleic acid stain, was added to the cells at 1 mM concentration and incubated for 5 min. Following a PBS wash, fading of the fluorescent signal was minimized by fixing the cells in Vectashield (Vector Laboratories). The cells were observed using a Leica DMRD fluorescence microscope, at 430 nm for DAPI, 488 nm for GFP, and 543 nm for rhodamine. Signals were recorded sequentially using PL APO x63 / 1.32 oil / PH3 */ 0.17/ D oil immersion objectives equipped with a filter for Nomarski. The VISIOLAB 200 program and a Sony 3CCD color video camera “Power HAD” were used for image processing.

## Supporting Information

Figure S1 Experimental Determination of the Rate of Polymerization Time versus extension traces recorded in a force-clamp operation mode at 12, 36, and 50.5 pN. Shown are zooms in the region where the transition (e.g., coverage of ssDNA by VirE2) occurs. At low forces (< ∼20 pN), the curves show first a fast decay that originates from multiple polymerization fronts running in parallel (Text S1, section: Rate of polymerization (experimental determination)]. This transition occurs so fast (up to 10 μm/s) that the feedback loop cannot follow in real time the polymerization. At high coverage, the probability of having multiple fronts is considerably reduced (due to the lack of free available VirE2 binding sites). As such, the time traces show clearly distinct linear regimes from which the polymerization rate originating from a single polymerization front can be determined (red line). At higher forces (36 and 50.5 pN) and even at low coverage, the probability of having polymerization fronts growing in parallel is greatly reduced due to the typical conformation of bare ssDNA found at high force (Text S1, section: Rate of polymerization (theory)]. In agreement with EM investigations, we found that the typical length of VirE2 domains is about tens of nanometers [18].
(690 KB PDF)

Figure S2 Graphical Representation of the Model for Equation 1 in Text S1 Describing the Binding of VirE2 on One Nucleotide (Located at Position 0) Rectangles indicate the location of the DNA phosphates. The arrow shows the direction of the applied force. Nucleotides already bound to VirE2 are overlaid with a grey rectangle.
(A) “Not-bound state”. α denotes the angle between the direction of the applied force (the long axis of the protein) and a ssDNA segment of length *L_SS_* (shown in bold) constrained at position 0. β denotes the angle between the direction of the applied force and an adjacent ssDNA segment (+1-+2).
(B) “Bound state”. α′ (β′) denotes the angle between the direction of the applied force and a ssDNA segment bound at position 0 and +1 (+1 and +2). Note that the contour length of a VirE2-bound ssDNA L_V_ corresponds to the projection of *L_SS_* along the direction of the applied force.
(257 KB PDF)

Figure S3 Estimation of the Rate of Polymerization *k* as a Function of the Applied Force See Text S1, section: Rate of polymerization [theory]
Experimental data points and curves obtained in a local model have been normalized to the rate at zero force. The enthalpy (left panel) or the Gibbs free energy (middle panel) was computed to estimate the force dependence of *k*. Lines are results from a local model calculation using known parameters for the base-to-base distance of bare and VirE2-bound ssDNA (0.7 and 0.41 nm, respectively [16,18]). Also shown is the influence of a change in the base-to-base distance of VirE2-bound ssDNA on the calculation (right panel). A value of 0.41 nm gives the best result.
(185 KB PDF)

Figure S4 Analysis of Individual Binding Events at High Force (A) Force-feedback experiment at ∼50 pN in the presence of VirE2 with lengths in nanometers (similar to Figure 1A). Inset: Trace between 340 and 420 nm length reduction, where steps from single or multiple VirE2 binding events are visible (space between arrows indicate the binding of 3 proteins, i.e., ∼10 nm). Length increase steps also occur and correspond to the unbinding of one or several monomers.
(B) Top: Probability density function (PDF, solid red line) calculated from the complete trace in (A). The probability density function (PDF) was determined by summing individual normal distributions with mean *x*_i_ and variance σ^2^ (where *x*_i_ denotes the experimentally measured filament length and σ = 2.2 nm for our apparatus) [36]. The distances between peaks (gray lines) are multiples of 3.35 nm (i.e., the ssDNA compaction produced by one VirE2 molecule on 19 nucleotides). This is shown in Figure S3 (bottom) with the PDF from 340 to 420 nm. Coloured bars indicate how many elementary compression steps occur in between each peak. Given the ssDNA base-to-base distance at ∼50 pN (0.57 nm; Figure S5) and the number of nucleotides bound per VirE2 monomer (19) [18], we found *L_V_* to be ∼0.395 nm (i.e*.,* 0.57 – (3.35/19). This value is in good agreement with that determined by EM (0.41 nm), assuming the ssDNA to lie concentrically within the helical protein filament. The result shown here is a first attempt to determine the base-to-base distance of VirE2-bound-ssDNA from single-molecule experiment. We emphasize that such experiments are extremely challenging due to the difficulty of keeping a fragile ssDNA molecule in flow at high tension for a few minutes.
(458 KB PDF)

Figure S5 Optical Tweezers Force Curves of DNA Typical force versus extension curves of dsDNA (green), and ssDNA (red) in assembly buffer (50 mM NaH_2_PO_4_ pH 8.0, 150 mM NaCl and 5% w/v glycerol). Curves are normalized to the contour length of ssDNA (assuming a base-to-base distance of 0.7 nm). The DNA base-to-base distance (obtained by multiplying the normalized extension by 0.7 nm) projected onto the direction of the applied force is shown at the top [16,20].
(321 KB PDF)

Figure S6 Distance-Clamp Experiment Trace Experimental time trace of a single ssDNA molecule upon VirE2 injection obtained in a distance-feedback optical tweezers operation mode. The distance is set at 0.14 normalized extension, corresponding to the normalized extension of ssDNA when a VirE2 helix is formed. The change in force measured upon injection of VirE2 proteins (up to ∼50 pN) is in good agreement with the values obtained from standard force *versus* elongation curves at an extension of 0.14 where no feedback is applied (Figure 3A).
(319 KB PDF)

Figure S7 Gel Shift Assay Showing the Cooperative Binding Mode of VirE2 on ssDNA Gel retardation analysis of reactions between a ∼170-bases-long ssDNA and VirE2. Radioactive ssDNA was incubated with VirE2 for 1 h and analyzed on a native 4% acrylamide gel. The fast migrating bands at the bottom represent the free ssDNA. Upon binding of VirE2, large nucleoprotein complexes formed, migrated slower and hence localized at the top of the gel. The binding of the proteins to ssDNA was cooperative, as hardly any intermediate ssDNA–protein complexes were detected.
(1 MB PDF)

Text S1 The Experimental and Theoretical Determination of the Polymerization Rate and the Mechanics of DNA-VirE2 Nucleoprotein Filaments (97 kB DOC)

## Abbreviations

BAP: 6 benzylaminopurine
ds: double-strand
EM: electron microscopy
FJC: freely jointed chain
GUS: β-glucuronidase
MS: Murashig and Skoog
ss: single-strand
T-DNA: transferred DNA
T4SS: Type IV secretion system
