# Supplementary figures and images for: VirE2: A Unique ssDNA-Compacting Molecular Machine
Source: PLoS Biol. 2008 Feb 26;6(2):e44. doi: 10.1371/journal.pbio.0060044 (PMC2253637; doi:10.1371/journal.pbio.0060044)

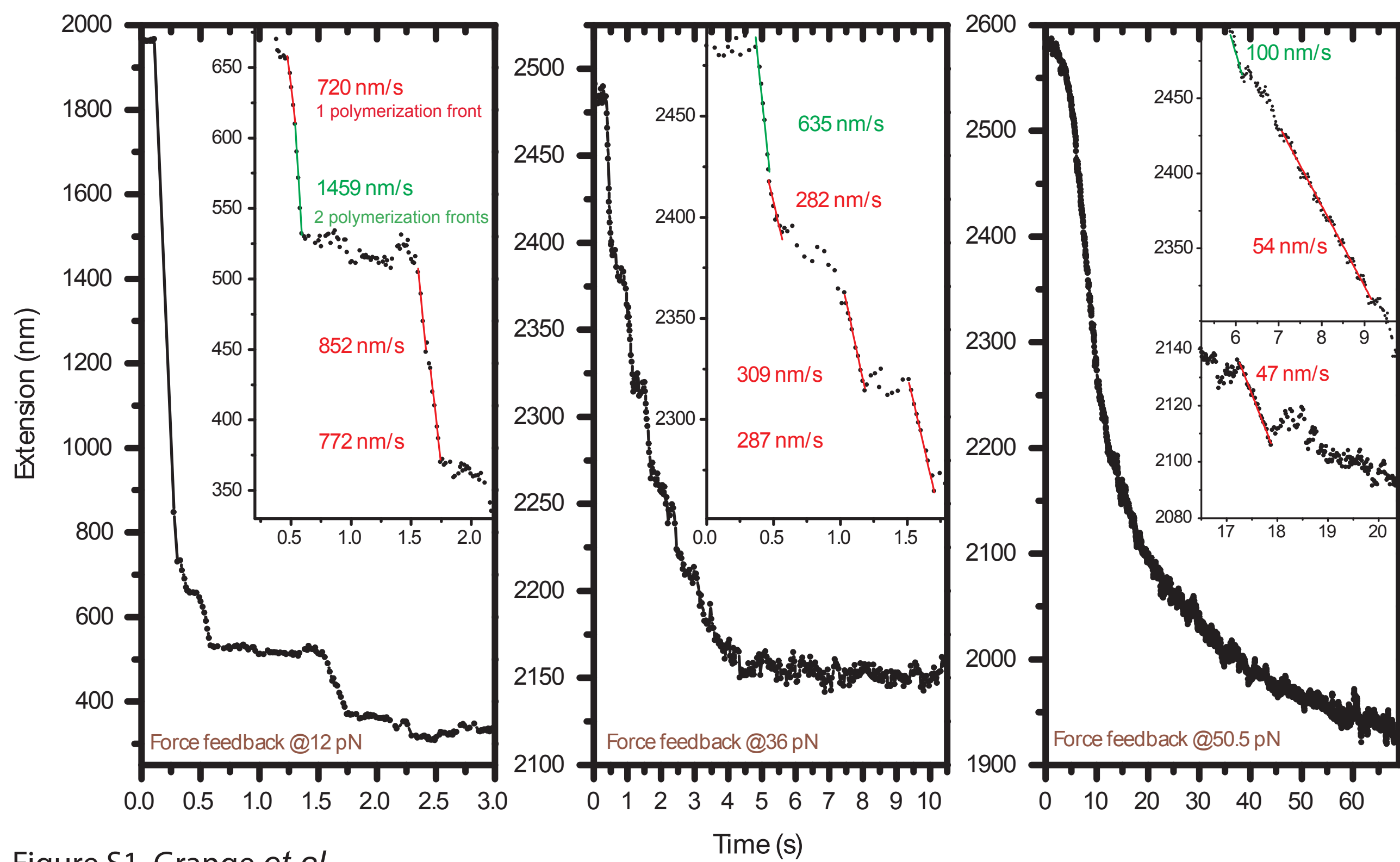

Figure S1. Grange *et al.*

Supplement: Figure S1 — Time versus extension traces recorded in a force-clamp operation mode at 12, 36, and 50.5 pN. Shown are zooms in the region where the transition (e.g., coverage of ssDNA by VirE2) occurs. At low forces (< ∼20 pN), the curves show first a fast decay that originates from multiple polymerization fronts running in parallel (Text S1, section: Rate of polymerization (experimental determination)]. This transition occurs so fast (up to 10 μm/s) that the feedback loop cannot follow in real time the polymerization. At high coverage, the probability of having multiple fronts is considerably reduced (due to the lack of free available VirE2 binding sites). As such, the time traces show clearly distinct linear regimes from which the polymerization rate originating from a single polymerization front can be determined (red line). At higher forces (36 and 50.5 pN) and even at low coverage, the probability of having polymerization fronts growing in parallel is greatly reduced due to the typical conformation of bare ssDNA found at high force (Text S1, section: Rate of polymerization (theory)]. In agreement with EM investigations, we found that the typical length of VirE2 domains is about tens of nanometers [18]. (690 KB PDF) [file pbio.0060044.sg001.pdf]

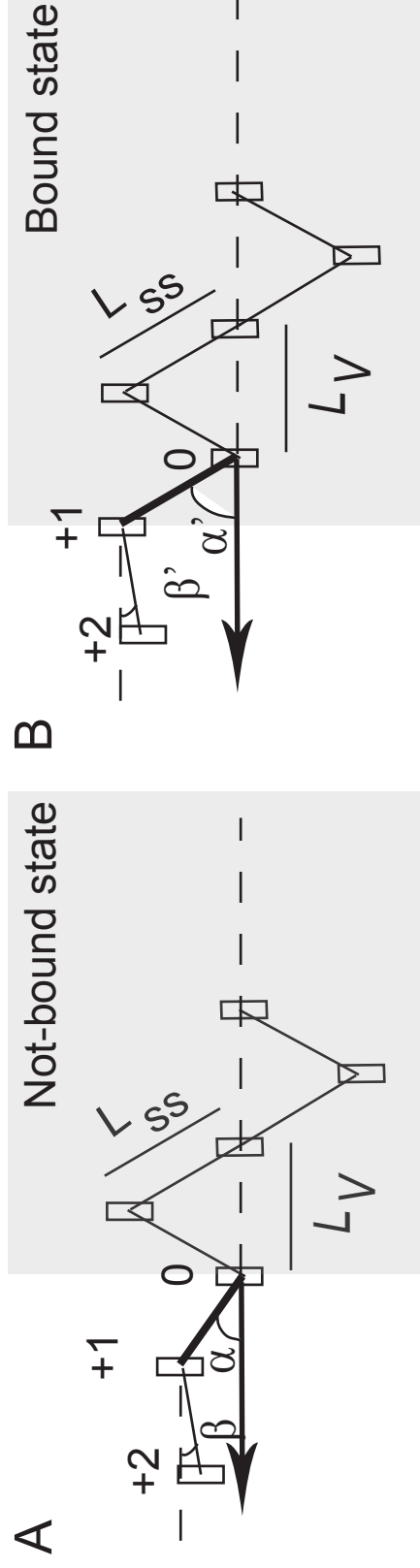

Figure S2. Grange *et al.*

Supplement: Figure S2 — Rectangles indicate the location of the DNA phosphates. The arrow shows the direction of the applied force. Nucleotides already bound to VirE2 are overlaid with a grey rectangle. (A) “Not-bound state”. α denotes the angle between the direction of the applied force (the long axis of the protein) and a ssDNA segment of length LSS (shown in bold) constrained at position 0. β denotes the angle between the direction of the applied force and an adjacent ssDNA segment (+1-+2). (B) “Bound state”. α′ (β′) denotes the angle between the direction of the applied force and a ssDNA segment bound at position 0 and +1 (+1 and +2). Note that the contour length of a VirE2-bound ssDNA LV corresponds to the projection of LSS along the direction of the applied force. (257 KB PDF) [file pbio.0060044.sg002.pdf]

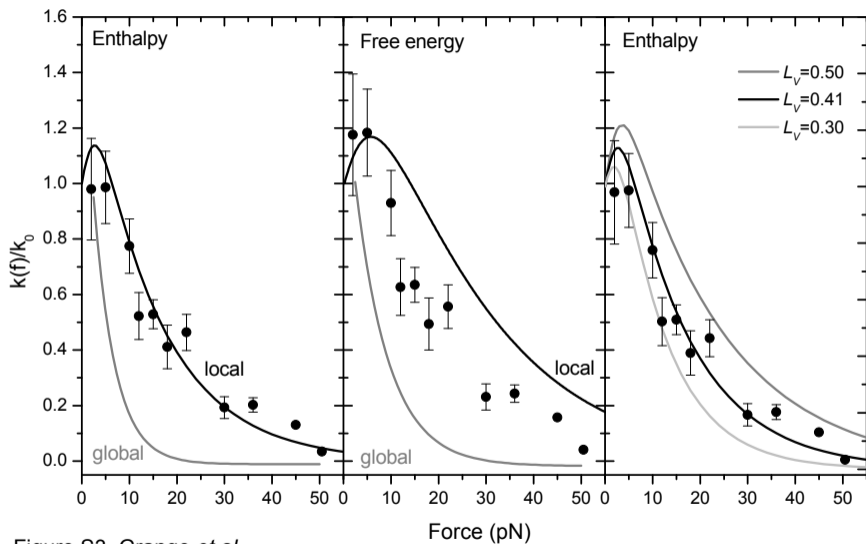

Figure S3. Grange *et al.*

Supplement: Figure S3 — See Text S1, section: Rate of polymerization [theory] Experimental data points and curves obtained in a local model have been normalized to the rate at zero force. The enthalpy (left panel) or the Gibbs free energy (middle panel) was computed to estimate the force dependence of k. Lines are results from a local model calculation using known parameters for the base-to-base distance of bare and VirE2-bound ssDNA (0.7 and 0.41 nm, respectively [16,18]). Also shown is the influence of a change in the base-to-base distance of VirE2-bound ssDNA on the calculation (right panel). A value of 0.41 nm gives the best result. (185 KB PDF) [file pbio.0060044.sg003.pdf]

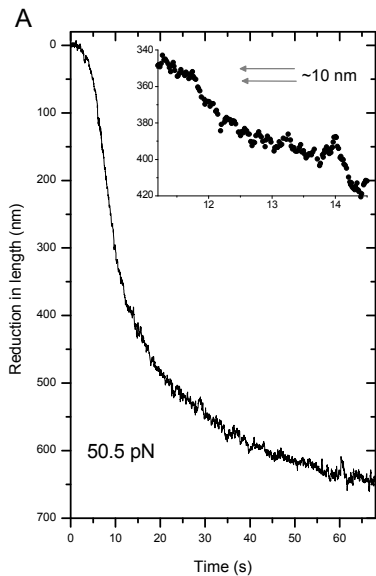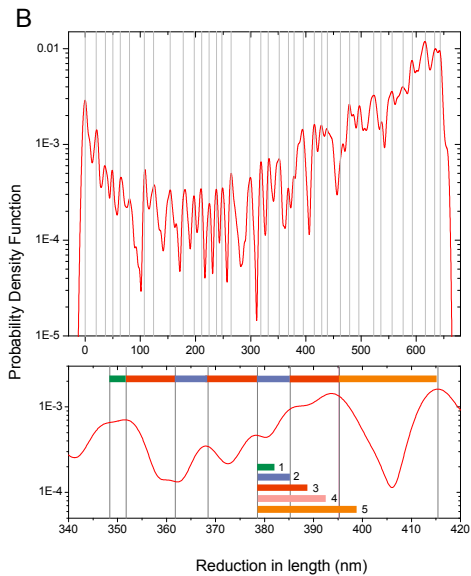

Figure S4. Grange et al.

Supplement: Figure S4 — (A) Force-feedback experiment at ∼50 pN in the presence of VirE2 with lengths in nanometers (similar to Figure 1A). Inset: Trace between 340 and 420 nm length reduction, where steps from single or multiple VirE2 binding events are visible (space between arrows indicate the binding of 3 proteins, i.e., ∼10 nm). Length increase steps also occur and correspond to the unbinding of one or several monomers. (B) Top: Probability density function (PDF, solid red line) calculated from the complete trace in (A). The probability density function (PDF) was determined by summing individual normal distributions with mean x i and variance σ2 (where x i denotes the experimentally measured filament length and σ = 2.2 nm for our apparatus) [36]. The distances between peaks (gray lines) are multiples of 3.35 nm (i.e., the ssDNA compaction produced by one VirE2 molecule on 19 nucleotides). This is shown in Figure S3 (bottom) with the PDF from 340 to 420 nm. Coloured bars indicate how many elementary compression steps occur in between each peak. Given the ssDNA base-to-base distance at ∼50 pN (0.57 nm; Figure S5) and the number of nucleotides bound per VirE2 monomer (19) [18], we found LV to be ∼0.395 nm (i.e., 0.57 – (3.35/19). This value is in good agreement with that determined by EM (0.41 nm), assuming the ssDNA to lie concentrically within the helical protein filament. The result shown here is a first attempt to determine the base-to-base distance of VirE2-bound-ssDNA from single-molecule experiment. We emphasize that such experiments are extremely challenging due to the difficulty of keeping a fragile ssDNA molecule in flow at high tension for a few minutes. (458 KB PDF) [file pbio.0060044.sg004.pdf]

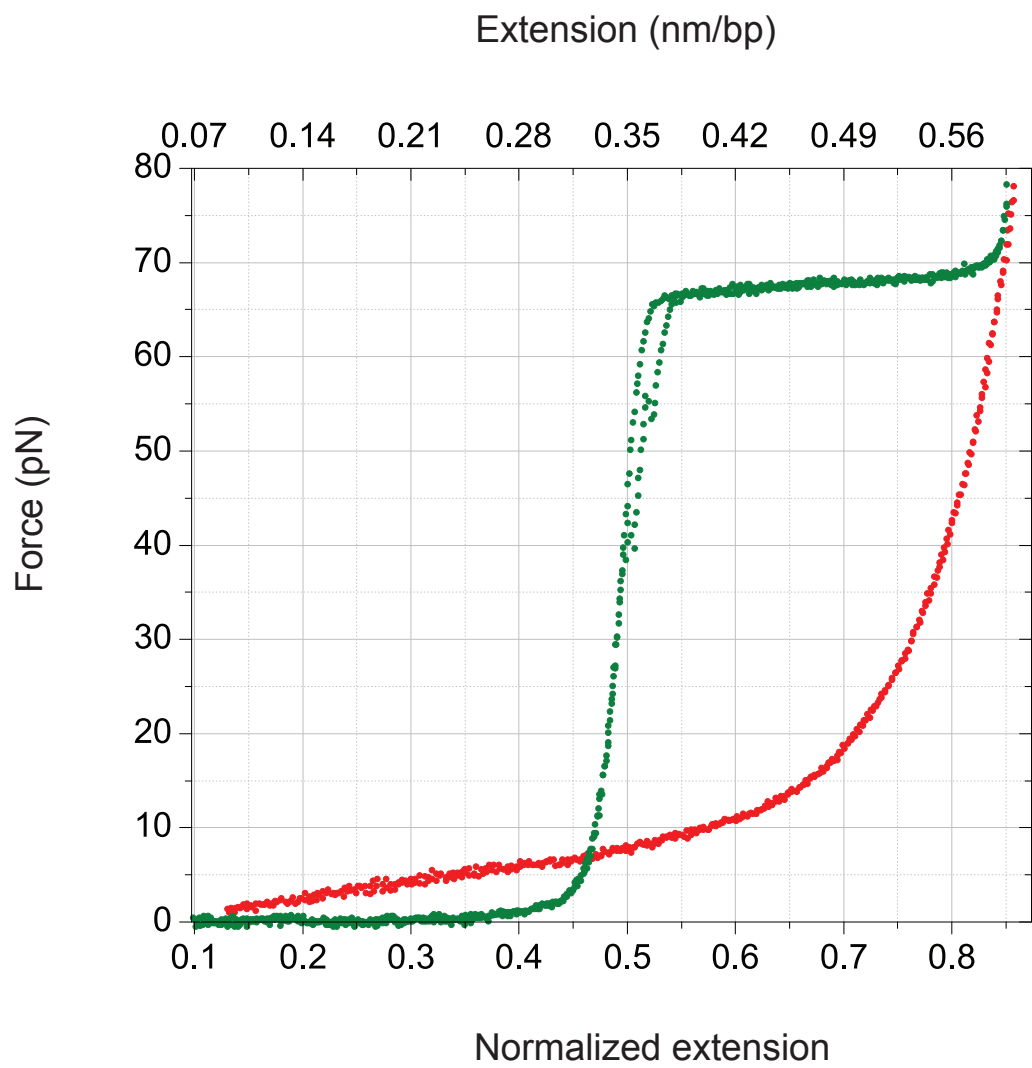

Figure S5. Grange *et al.*

Supplement: Figure S5 — Typical force versus extension curves of dsDNA (green), and ssDNA (red) in assembly buffer (50 mM NaH2PO4 pH 8.0, 150 mM NaCl and 5% w/v glycerol). Curves are normalized to the contour length of ssDNA (assuming a base-to-base distance of 0.7 nm). The DNA base-to-base distance (obtained by multiplying the normalized extension by 0.7 nm) projected onto the direction of the applied force is shown at the top [16,20]. (321 KB PDF) [file pbio.0060044.sg005.pdf]

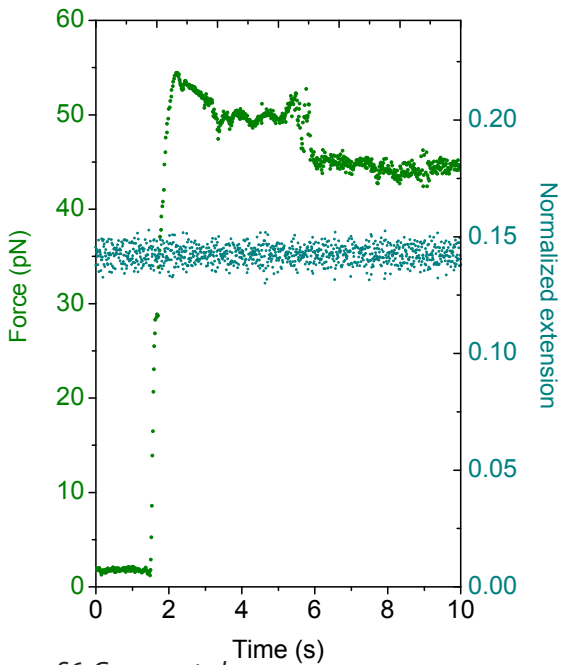

Figure S6. Grange *et al.*

Supplement: Figure S6 — Experimental time trace of a single ssDNA molecule upon VirE2 injection obtained in a distance-feedback optical tweezers operation mode. The distance is set at 0.14 normalized extension, corresponding to the normalized extension of ssDNA when a VirE2 helix is formed. The change in force measured upon injection of VirE2 proteins (up to ∼50 pN) is in good agreement with the values obtained from standard force versus elongation curves at an extension of 0.14 where no feedback is applied (Figure 3A). (319 KB PDF) [file pbio.0060044.sg006.pdf]

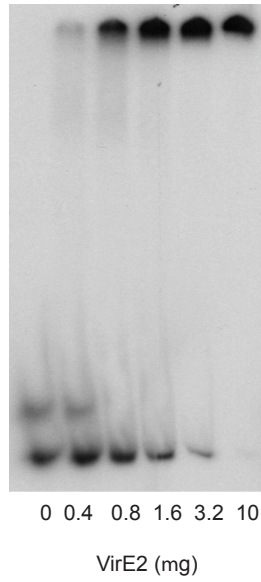

Figure S7. Grange *et al.*

Supplement: Figure S7 — Gel retardation analysis of reactions between a ∼170-bases-long ssDNA and VirE2. Radioactive ssDNA was incubated with VirE2 for 1 h and analyzed on a native 4% acrylamide gel. The fast migrating bands at the bottom represent the free ssDNA. Upon binding of VirE2, large nucleoprotein complexes formed, migrated slower and hence localized at the top of the gel. The binding of the proteins to ssDNA was cooperative, as hardly any intermediate ssDNA–protein complexes were detected. (1 MB PDF) [file pbio.0060044.sg007.pdf]
