# Supplementary material for: VirE2: A Unique ssDNA-Compacting Molecular Machine
Source: PLoS Biol. 2008 Feb 26;6(2):e44. doi: 10.1371/journal.pbio.0060044 (PMC2253637; doi:10.1371/journal.pbio.0060044)
Supplement: Text S1 — (97 kB DOC) [file pbio.0060044.sd001.doc]

# VirE2: a unique ssDNA compacting molecular machine

Wilfried Grange, Myriam Duckely, Sudhir Hustle, Susan Jacob, Andreas Engel and Martin Hegner

**Supplementary Text**

**Rate of polymerization (experimental determination)**

Here we present the analysis we have used to determine the polymerization rate originating from a single nucleation site. This analysis allows an accurate comparison to me made between the experimentally determined rates and the theoretical description (the local model) introduced in the next section of this Supplementary Text.

As illustrated in Figures 1A and S1, time traces recorded in the force-clamp operation mode show two distinct regimes at low forces (< ~ 20 pN). First a rapid decay (for which no intermediate points are recorded) that yields a change in extension of about 75% (1231 nm in 171 ms at 12 pN, Figure S1) followed by a slower regime that leads to a complete coverage. As described in Materials and Methods section (Force-clamp operation mode), the lack of intermediate points in the fast regime is attributed to the fact that binding of VirE2 on ssDNA produces changes in force > 0.7 pN within 6.7 ms at low coverage. In that case, the feedback loop (working at a frequency comparable to other state-of-the-art optical tweezers devices [1]) will have some delay and the molecule in the clamp will not experience a true constant-force regime. The lack of experimental points in the fast decay part of the time trace does not allow an accurate determination of the polymerization rates (originating from a single nucleation site). Most probably, this fast decay in length may be the result of many VirE2 nucleation sites that form immediately after addition of proteins, which subsequently leads to the progressive coverage of ssDNA by VirE2 from different locations on ssDNA. This behavior (multiple nucleation sites) is common and has recently been observed in the case of Rad51 that also polymerize on ssDNA [2]. It is highly probable, since the VirE2 concentration used in our experiment is high (20 μg/ml), and because the conformation of bare ssDNA at low forces almost matches that found in VirE2-bound ssDNA. Indeed, and as shown in the following section [“Rate of polymerization (theory)”, first paragraph], the average angular fluctuations of bare ssDNA yields a base-to-base distance of about 0.4 nm (projected along the direction of the applied force at low force, e.g. ~ 5 pN) that is similar to that found when VirE2 binds ssDNA.

Once a high VirE2 coverage on ssDNA is obtained (75 % or larger), only a few binding sites are still available for VirE2. As such, the probability of having many nucleation sites forming at the same time (say in 100 ms) is dramatically reduced. This is exactly what is observed in our experiment. As seen in Figure S1 (left panel, 12 pN), the time traces obtained in a force-clamp operation mode show at high coverage several distinct linear regimes. From a detailed analysis of the time trace (isolating parts in the time trace that correspond to a distinct growth velocity without any inflection points), we can determine the polymerization rate that corresponds to a single nucleation event. At 12 pN (Figure S1; left panel, inset), this yields a value of about 750 nm/s (red line). Of course, there can still be discrete numbers of polymerization fronts growing in parallel. For example, a linear regime having a slope of 1459 nm/s is observed (green line) and should be attributed to the presence of two different fronts (originating from distinct nucleation events). It is interesting to mention that the length of VirE2 domains (originating from a single nucleation event) is about a few tens of nanometers, in good agreement with high resolution EM observations [3].

At higher forces (e.g. 36 and 50.5 pN, Figure S1), the probability of having polymerization fronts growing in parallel is greatly reduced because the conformation of bare ssDNA does not match that found in the constrained VirE2-ssDNA filament (section [“Rate of polymerization (theory)”]. Since the polymerization rate originating from a single nucleation site is also considerably reduced at higher forces (Figure 1B, blueline), the feedback loop is fast enough to follow in real time the polymerization of VirE2 on ssDNA (intermediate points are recorded). Here, the probability of having different fronts running in parallel, although small, is not negligible and therefore we still observe linear regimes that should be attributed to the presence of two different polymerization fronts running in parallel (green lines).

**Rate of polymerization (theory)**

**1. Local model**

*k(f)* was modelled using an Arrhenius law [4]:

$k(f)=k_{0}\exp(-<w(f)>/k_{B}T)$, where *<w(f)>* represents the time average of the work produced during binding of the protein to one nucleotide (Figure S2):

$<w(f)>=f\left(L_{SS}\left\langle\cos\alpha\right\rangle-L_{SS}\left\langle\cos\alpha'\right\rangle\right)-f\left(L_{SS}\left\langle\cos\beta\right\rangle-L_{SS}\left\langle\cos\beta'\right\rangle\right)$ Eq. 1

where *L_SS_* denotes the contour length of free ssDNA. Assuming that the adjacent nucleotide +2 is unhindered by the protein binding on segment +1-0 and that the segment +1-0 is firmly constrained in the bound state (Figure S2), we have: β=β’ and *L_SS_<*cosα’>=*L_V_* (*L_V_* represents the local contour length of VirE2-bound ssDNA), respectively.

Under such approximation, the time-averaged of *w(f)* reads:

$<w(f)>=f\left(L_{SS}\left\langle\cos\alpha\right\rangle-L_{V}\right)$ Eq. 2

In freely jointed chain (FJC) model, the angular fluctuations of a free ssDNA molecule follow the Langevin formula. This gives:

$<w(f)>=fL_{SS}\left[\left(\coth\frac{2A_{SS}f}{k_{B}T}-\frac{k_{B}T}{2A_{SS}f}\right)-\frac{L_{V}}{L_{SS}}\right]$ Eq. 3

For the persistence length *A_ss_* and the local contour length *L_ss_* of free ssDNA we used: *A_ss_*=0.75 nm [5] and *L_ss_*=0.7 nm [6], respectively. The value of the local contour length of ssDNA bound to VirE2, *L_V_*=0.41 nm, originates from the 3D reconstruction of the helical ssDNA-VirE2 filaments by EM [3]. Note that correcting the FJC model for the finite elasticity of ssDNA (*i.e.* multiplying the left term in the brackets of Eq. 1 by (1+f/S), where S denotes the stretch modulus [5]) does not notably change the overall shape of the curve. The good agreement between the experimental force-feedback data and the local model as well as the results of our gel-shift assay (Figure 2B), indicate that VirE2 can bind individual bases of ssDNA in a zipper-like motion. As seen in Figures 1B and S3, the force dependence of the polymerization shows a maximum at about 6 pN. This is attributed to the fact that the average value of the angular fluctuations of bare ssDNA yields a base-to-base distance (projected along the direction of the applied force) similar to that found in the constrained VirE2-ssDNA filament ($L_{SS}\left\langle\cos\alpha\right\rangle$= 0.41 nm at 6.3 pN, in good agreement with EM studies).

**2. Global model**

Although we have computed the averaged work *<w(f)>* by analyzing the local conformation of ssDNA on the active binding site (local model), a global model is often used to explain the dependence of the rate of polymerization of motor enzymes (see for instance [7,8]). In a global model, the work is directly estimated from the global force *versus* extension curves [9]:

$\Delta g(f)=nf(x_{ss}-x_{VirE2})-T\Delta S(f)$ Eq. 4

where $nf(x_{ss}-x_{VirE2})$represents the work to convert *n* bases from the ssDNA geometry to the VirE2-bound geometry. *x_VirE2_* (*x_SS_*) are the extensions per base (measured along the direction of the applied force) directly determined from the force *versus* extension curves. Although it has been widely used, the global model ignores the specific geometry and interactions of the DNA at the active site. For our experiments, the force-curves only show the global structure (*i.e.* the re-arrangement in a helical structure) of the VirE2-ssDNA complex ignoring the local binding mode of VirE2. As seen in Figure S3, the global model fails to give a good estimate of the force-dependence of *k* (the final global stretching curves are mainly determined by the helical structure of the filament). We therefore propose that the local model gives a better description of the experimental observations.

**3. Enthalpy *versus* free energy**

In Eq. 1, the enthalpy rather than the free energy was used to determine *k(f)*. We point out that the literature is still inconsistent in the usage of enthalpy and free energy [9]. For this reason, we show the dependence of *k(f)* using both models (enthalpy and free energy) in Figure S3 and note that taking the enthalpy difference in Eq. 1 gives slightly better results.

**Length reduction upon protein binding**

The paper [3] shows a 6.8 fold (from 2900 to 425 nm) reduction in length of their M13 ssDNA substrate assuming a canonical length of 0.4 nm per base for bare ssDNA. Using a base-to-base distance of 0.7 nm for bare ssDNA [6], the expected compaction of the 7249 bases M13 ssDNA is 7249*0.7/425=11.9 with respect to ssDNA, which is comparable to our findings.

**Mechanical properties of ssDNA-VirE2 filaments: Helix model**

The value of the persistence length *A* of a protein helix (with pitch *P*, Young modulus *E*, moment of inertia *I* and inclination angle α) can be determined theoretically. Using a procedure similar to that presented in [10], we get:

$\frac{A}{k_{B}T}=\frac{\pi PEI}{\left[\left(2\pi R\right)^{2}+P^{2}\right]^{0.5}}\left(2\left(2+\sigma\right)\cos\alpha+\pi\sin\alpha\right)^{-1}$ Eq.5

For a cylinder with radius *r*, *I* is equal to πr^4^/4. From [11], we have *P*=5.15 nm, *I*=17.6 nm^4^, r=2.1 nm and α=9.1 degrees. Using typical values of *E*=(0.3-6)X10^3^ pN.nm ^–2^ [11] and a Poisson ratio of σ=0.5 for a protein, we find *A*=(110-2200) nm.

Similarly, the stretch modulus *S* of the protein helix can be obtained using:

$S=\frac{PEI}{R^{2}\left[\left(2\pi R\right)^{2}+P^{2}\right]^{0.5}}\left(\sin^{2}\alpha+(1+\sigma)\cos^{2}\alpha\right)^{-1}$ Eq.6

For VirE2 filaments we found *S*=(25-320) pN.

The procedure we used yields a lower estimate of *A* and *S*. First, interactions between proteins and the DNA template are not taken into account. Second, the 3D structure of the helical VirE2-ssDNA filament [3] shows axial inter-molecular interactions between adjacent protein turns (Figure 2A, circles). These interactions are likely to stiffen the mechanical arrangement of the complex, but are neglected in the present calculation. Note that such a helical model gives a good estimate of the persistence length, when no axial interactions are found (e.g. RecA binding to ssDNA [10]).

**References**

1. Wen JD, Manosas M, Li PT, Smith SB, Bustamante C, et al. (2007) Force unfolding kinetics of RNA using optical tweezers. I. Effects of experimental variables on measured results. Biophys J 92: 2996-3009.

2. Mine J, Disseau L, Takahashi M, Cappello G, Dutreix M, et al. (2007) Real-time measurements of the nucleation, growth and dissociation of single Rad51 DNA nucleoprotein filaments. Nucleic Acids Res.

3. Abu-Arish A, Frenkiel-Krispin D, Fricke T, Tzfira T, Citovsky V, et al. (2004) Three-dimensional reconstruction of Agrobacterium VirE2 protein with single-stranded DNA. J Biol Chem 279: 25359-25363.

4. Goel A, Frank-Kamenetskii MD, Ellenberger T, Herschbach D (2001) Tuning DNA "strings": modulating the rate of DNA replication with mechanical tension. Proc Natl Acad Sci U S A 98: 8485-8489.

5. Smith SB, Cui Y, Bustamante C (1996) Overstretching B-DNA: the elastic response of individual double-stranded and single-stranded DNA molecules. Science 271: 795-799.

6. Saenger W (1984) Principles of Nucleic Acid Structure. Berlin: Springer.

7. Wuite GJ, Smith SB, Young M, Keller D, Bustamante C (2000) Single-molecule studies of the effect of template tension on T7 DNA polymerase activity. Nature 404: 103-106.

8. Maier B, Bensimon D, Croquette V (2000) Replication by a single DNA polymerase of a stretched single-stranded DNA. Proc Natl Acad Sci U S A 97: 12002-12007.

9. Andricioaei I, Goel A, Herschbach D, Karplus M (2004) Dependence of DNA polymerase replication rate on external forces: a model based on molecular dynamics simulations. Biophys J 87: 1478-1497.

10. Hegner M, Smith SB, Bustamante C (1999) Polymerization and mechanical properties of single RecA-DNA filaments. Proc Natl Acad Sci U S A 96: 10109-10114.

11. Morozov VN, Morozova TY (1981) Viscoelastic properties of protein crystals: triclinic crystals of hen egg white lysozyme in different conditions. Biopolymers 20: 451-467.
